# Tracking axon initial segment plasticity using high-density microelectrode arrays: A computational study

**DOI:** 10.3389/fninf.2022.957255

**Published:** 2022-10-03

**Authors:** Sreedhar S. Kumar, Tobias Gänswein, Alessio P. Buccino, Xiaohan Xue, Julian Bartram, Vishalini Emmenegger, Andreas Hierlemann

**Affiliations:** Bio Engineering Laboratory, Department of Biosystems Science and Engineering, ETH Zurich, Basel, Switzerland

**Keywords:** homeostatic plasticity, AIS plasticity, HD-MEAs, biophysical modeling, random forest classifier, neighborhood components analysis (NCA), LTI systems, wide neural networks

## Abstract

Despite being composed of highly plastic neurons with extensive positive feedback, the nervous system maintains stable overall function. To keep activity within bounds, it relies on a set of negative feedback mechanisms that can induce stabilizing adjustments and that are collectively termed “homeostatic plasticity.” Recently, a highly excitable microdomain, located at the proximal end of the axon—the axon initial segment (AIS)—was found to exhibit structural modifications in response to activity perturbations. Though AIS plasticity appears to serve a homeostatic purpose, many aspects governing its expression and its functional role in regulating neuronal excitability remain elusive. A central challenge in studying the phenomenon is the rich heterogeneity of its expression (distal/proximal relocation, shortening, lengthening) and the variability of its functional role. A potential solution is to track AISs of a large number of neurons over time and attempt to induce structural plasticity in them. To this end, a promising approach is to use extracellular electrophysiological readouts to track a large number of neurons at high spatiotemporal resolution by means of high-density microelectrode arrays (HD-MEAs). However, an analysis framework that reliably identifies specific activity signatures that uniquely map on to underlying microstructural changes is missing. In this study, we assessed the feasibility of such a task and used the distal relocation of the AIS as an exemplary problem. We used sophisticated computational models to systematically explore the relationship between incremental changes in AIS positions and the specific consequences observed in simulated extracellular field potentials. An ensemble of feature changes in the extracellular fields that reliably characterize AIS plasticity was identified. We trained models that could detect these signatures with remarkable accuracy. Based on these findings, we propose a hybrid analysis framework that could potentially enable high-throughput experimental studies of activity-dependent AIS plasticity using HD-MEAs.

## 1. Introduction

Neurons in the brain are highly adaptive and alter their behavior in response to learning and development-related changes. Yet, the nervous system is able to maintain stable overall function. The balance between flexibility and stability is maintained by an array of homeostatic plasticity mechanisms that allow individual neurons to regulate their synaptic inputs and/or intrinsic excitability (Turrigiano and Nelson, 2004; Turrigiano, 2011; Yin and Yuan, 2015).

Recent studies have proposed that one of the ways in which neurons regulate their intrinsic excitability may involve activity-dependent structural modulation of a specialized microdomain called the axon initial segment (AIS; Kuba et al. 2010; Grubb and Burrone 2010; Lezmy et al. 2017). The AIS is a highly excitable region of the neuron, being densely populated by voltage-gated sodium and potassium channels (Kole et al., 2008).

Structural plasticity, observed at the AIS, includes changes in AIS length (Evans et al., 2013) and position (Grubb and Burrone, 2010; Lezmy et al., 2017) along the proximal axon. These changes were observed in response to activity perturbations in the network: for example, chronic depolarization of cultured hippocampal neurons was found to lead to a distal relocation of the AIS and was accompanied by a loss of intrinsic excitability (Grubb and Burrone, 2010). Conversely, following a loss of cochlear afferent innervation, increased AIS lengths and higher levels of intrinsic excitability were observed in neurons in the nucleus magnocellularis of birds *in vivo* (Kuba et al., 2010).

Structural modifications of the AIS appear to help restore the neuron to excitability levels prior to the activity perturbation (Lezmy et al., 2017). It is hence thought to serve a homeostatic function. Disruption of the molecular organization of the AIS could, thus, lead to neurological abnormalities. Indeed, several neurological disorders, such as epilepsy, Alzheimer's disease, neuroinflammatory or demyelination pathologies, bipolar disorder and schizophrenia are thought to impact AIS function (Buffington and Rasband, 2011; Hsu et al., 2014; Sun et al., 2014; Hamada and Kole, 2015). Hence, an understanding of the principles underlying AIS plasticity may help to better understand the pathological deficits resulting from AIS dysfunction and to guide the development of effective therapeutic interventions.

However, many aspects of AIS plasticity and its functional role in regulating neuronal and network excitability remain elusive. One of the challenges is the rich heterogeneity in its expression and the influence of a number of uncontrollable factors including neuron types, morphological parameters like soma size, axon diameter, and baseline AIS geometry (Gulledge and Bravo, 2016). Hence, there tends to be wide variability in the expression of the phenomenon within a network.

Current approaches to investigate structural plasticity of the AIS have several important limitations. Most studies compare the statistics of the AIS's structural metrics between snapshots of control networks and networks that have been perturbed and allowed to homeostasize over a fixed duration (Dumitrescu et al., 2016). The use of summary measures fails to account for neuronal heterogeneity present in each population—in terms of cell types, structural parameters of the cell, and initial AIS locations—and obscures the contribution of individual neurons to network effects (Gulledge and Bravo, 2016; Wefelmeyer et al., 2016). Moreover, histological studies generally target structural components of the AIS and not the channels themselves and may thus be inadequate to capture changes in the length and location of the “functional AIS.” The whole-cell patch-clamp technology, prevailingly used to study the functional impacts of AIS plasticity, does not permit long-term tracking of the same neuron due to its invasiveness. Long-term simultaneous access to both, subcellular structures of multiple neurons and activity in the entire network, is desirable to extract a multiscale, time-resolved picture of the phenomenon.

High-density microelectrode arrays (HD-MEAs), feature large enough spatiotemporal resolution and could be a powerful tool for high-throughput investigations of AIS plasticity. The recorded extracellular signals at each electrode represent the superposition of the activity of multiple neurons in each electrode's neighborhood. These complex signals need to be post-processed and assigned to putative signal-generating neurons by using sophisticated data-analysis techniques to extract neurophysiologically relevant information. A commonly used technique, called ‘spike sorting', aims to infer the spiking activity of single neurons from the recorded signals (Buccino et al., 2022a). At the network scale, spike sorting not only provides a readout of the spiking activity of several hundreds of neurons in the network but also enables to extract average extracellular footprints (templates) corresponding to each putative unit (inferred neuron).

The AIS is thought to be the dominant contributor to a neuron's extracellular footprint, i.e., the distribution of the extracellular electrical potentials of the neuron across the array electrodes during action potential generation (Bakkum et al., 2019). Therefore, we hypothesize that plastic changes of the AIS will yield complex but systematic changes in a neuron's extracellular footprints. Analyzing long-term trends in the extracellular footprint could pave the way to track AIS plasticity simultaneously in multiple neurons.

However, reliably inferring subtle microstructural changes from long-term changes in extracellular footprints is a formidable data-analysis challenge. Changes in the extracellular footprint, arising from structural modifications of the AIS, are difficult to predict without prior knowledge of the morphology of the proximal axon and its distance and orientation relative to the electrodes.

Further, factors like neuronal drift and movements relative to the electrodes may also lead to extracellular footprint changes that are potentially misleading. Neurons in primary dissociated cultures *in vitro* exhibit activity-dependent migration throughout development. Such “drifts” are dominant at early stages of development but tend to decay during development (Sun et al., 2011; Okujeni and Egert, 2019). Apart from spontaneous drifts, we presume that inadvertent shifts in cell positions (“movements”) may be induced by external perturbations, for example, during exchange of growth media or during experimenter handling. Together, drifts and movements presumably contribute to the variability of extracellular electrical footprints measured over long durations. In extreme cases, footprint changes could confound the detection of AIS plasticity based on extracellular recordings.

Hence, it is hitherto unclear if it would be feasible to infer structural plasticity from extracellular footprints. Sophisticated computational models could help to address this open issue by systematically elucidating the association between neuronal microstructures and measured extracellular field potentials.

In this study, we characterized changes in the extracellular electrical-potential landscape arising from structural changes of the AIS. We specifically focused on distal relocation—the phenomenon of spatial translocation of the AIS to a distal domain farther from the soma—and explored the feasibility of reliably inferring this structural modification from simulated extracellular electrical potentials. To this end, we used detailed multi-compartment models of single neurons with varying morphologies: a minimal ball-and-stick model and two different detailed morphology models.

We equipped a section of the proximal axon of each neuron with AIS-specific ion-channel conductances, whose positions along the axon were systematically altered. We generated action potentials by stimulating each neuron model with constant currents and generated the corresponding extracellular signals for the HD-MEA probe model using the LFPy package (Hagen et al., 2018).

Our results suggest that microstructural changes in the AIS may be reliably inferred by tracking long-term changes in extracellular footprints. Based on our *in silico* characterization, we propose two hybrid methods (combining simulations and experimental data) that could potentially facilitate high-throughput experimental studies of activity-dependent AIS plasticity. Although further studies are necessary to rigorously validate our findings using ground-truth experiments, our work demonstrates how computational modeling could be a valuable tool to augment the interpretational value of extracellular electrophysiology.

## 2. Methods

### 2.1. Neuron models

**Ball-and-stick-model:** A minimal biophysical ball-and-stick model was created with the following geometry: a 1 mm-long cylindrical dendrite with a diameter of 2 μm, a spherical soma with a diameter of 16 μm, and a thin cylindrical axon 1 mm long and 1 μm in diameter. A proximal section of the axon of 30 μm length was designated as the AIS. The distance between the soma and the proximal AIS was systematically varied between 5 and 40 μm.

**Detailed morphology-1:** For the detailed morphology model-1 (DM1) we built a conductance-based detailed multi-compartment neuron model whose morphology was reconstructed from an *in vitro* labeled L5b rat neocortical pyramidal neuron and was reported in Hay et al. (2011). In short, the neuron in a brain slice was filled with biocytin and reconstructed under a light microscope with the Neurolucida software (MicroBrightField, Magdeburg, Germany). To insert an AIS to the morphology model, we removed the original axon, connected a new segment representing the axon hillock to the soma, connected the AIS segment to the axon hillock and finally connected a straight cylindrical axon of length 500 μm to the distal end of the AIS.

**Detailed morphology-2:** For the detailed morphology model-2 (DM2) we built a conductance-based detailed multi-compartment neuron model whose morphology was reconstructed from a neuron in a primary rat dissociated cortical culture, prepared as described in Xue et al. (2022). All experimental protocols involving animals were approved by the Basel-Stadt veterinary office according to Swiss federal laws on animal welfare, and were carried out in accordance with the approved guidelines.

An individual neuron was filled with the fluorescent dye Alexa Fluor 594 via a patch-clamp microelectrode at 22 days *in vitro* (DIV). To achieve this, we used a patch-clamp setup that comprised a MultiClamp 700B amplifier (Axon Instruments) and an Axon Digidata 1440A (Axon Instruments). The cell culture was perfused with BrainPhys^TM^ Neuronal Medium (Stem Cell Technologies), heated to approximately 32–34°C throughout the experiment. The internal patch-clamp solution contained (in mM): 110 potassium-gluconate, 10 KCl, 10 Hepes, 4 MgATP, 0.3 GTP, 10 phosphocreatine, (pH 7.2-7.3; 280-290 mOsmol/l). On the day of the experiment, Alexa Fluor 594 (50 μM) was added to the internal solution. The neuron was patched with a standard borosilicate glass micropipette (4–5 MΩ) and loaded for more than 30 min in whole-cell current-clamp mode.

Subsequently, the culture was transferred to an upright confocal microscope (Nikon NiE) equipped with a spinning disk scan head (Yokogawa W1). An excitation laser of 561 nm and an emission filter of 609/54 nm was used for imaging. The neuron was imaged at 60x magnification (60x/1.00 NA water-objective, Nikon) with 6 x 4 tiles covering the entire morphology. For each tile, a z-stack (66 z images) was acquired with a 0.4 μm z-step. The x-y pixel size of the images was 112.55 nm.

Huygens Professional^1^ (version 21.10, Scientific Volume Imaging, The Netherlands) was used to perform the image deconvolution and stitching. Specifically, images were first deconvolved using the Classic Maximum Likelihood Estimation algorithm, with SNR:12 and 40 iterations. Subsequently, the deconvolved images were stitched with an overlap of 10%, using the circular vignetting correction model. The SNT plugin in Fiji was employed to reconstruct the 3D morphology of the neuron (Arshadi et al., 2021). All branches originating from the soma were merged into the same root to indicate the soma position with a single point. We then tagged the neurites as “soma,” “dend,” and “axon” accordingly. We preprocessed the raw morphology with a custom Python function that interpolated missing radii (below or equal to 0.1 μm) and smoothed the radii of each path with a 15-sample moving average. We followed a similar procedure as in DM1 to insert an AIS into the morphology. However, to accommodate the orientation of the axon hillock in this specific neuron, the axonal cylinder with the AIS was inserted at an orientation of −115.6°. The angle was computed based on an assessment of the morphology of the proximal axon.

**Biophysical Parameters:** We used the same biophysical parameters for our three neuronal models. Ion channel densities, conductances, and kinetics were set as in the study by Hallermann et al. (2012).

Briefly, sodium (*g*_*Na*_) and potassium (*g*_*K*_) channel current densities were set to 50 pS/μm^2^ and 100 pS/μm^2^ at the soma. The AIS was assigned high channel densities: *g*_*Na*_ =  7,000 pS/μm^2^ and *g*_*K*_ =  2,000 pS/μm^2^. Their equilibrium potentials were set to 55 and -98 mV respectively. In addition to these sodium and potassium channel models, both soma and AIS compartments contained low voltage-activated non-inactivating *K*_*v*_7 channels at a density of 1 and 7 pS/μm^2^. In the dendrites, *g*_*Na*_ was set to 20 pS/μm^2^, *g*_*k*_ to 0.3 pS/μm^2^, and *g*_*K*_*v*_7_ to 1 pS/μm^2^.

### 2.2. MEA model

**Figure 1:**
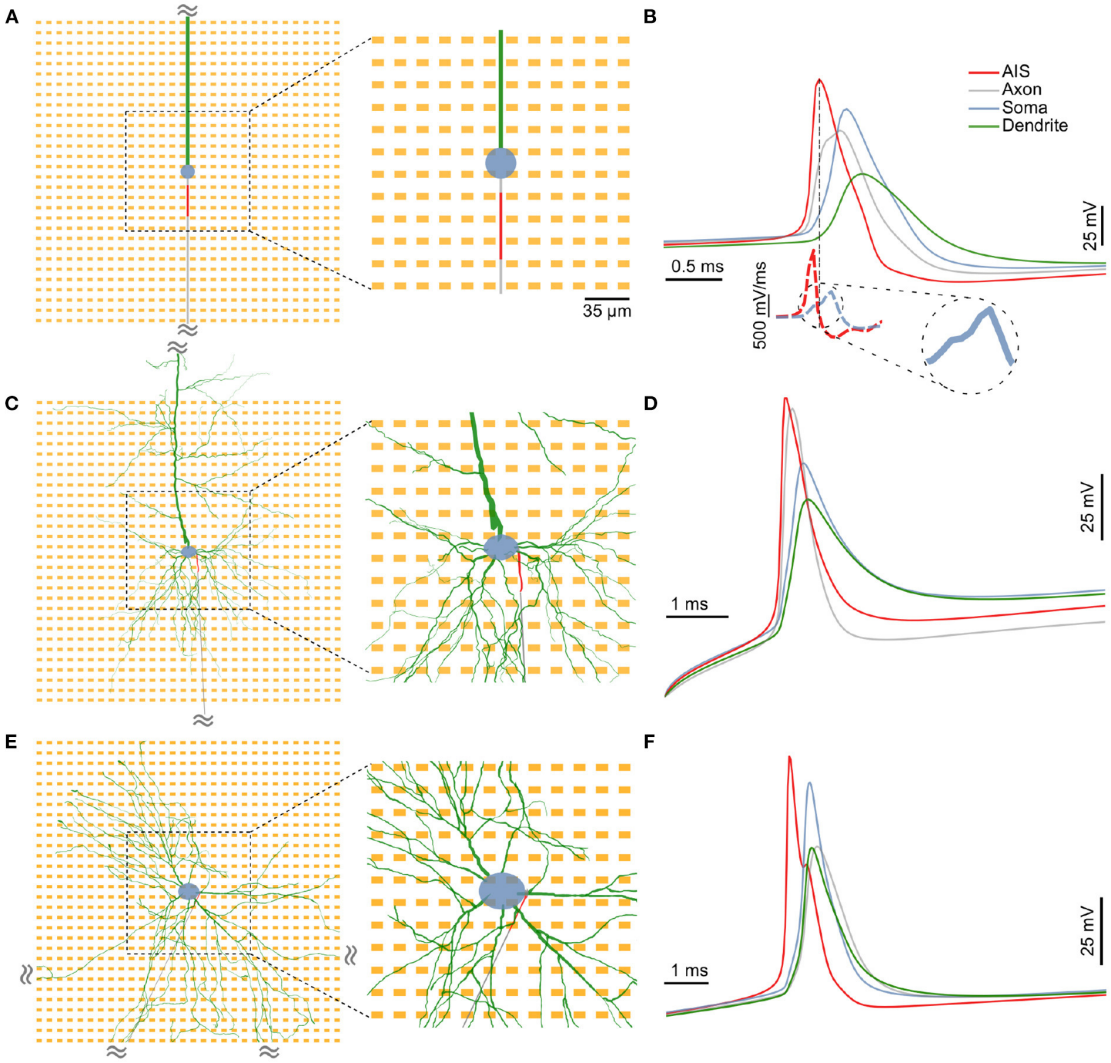
The three distinct neuron models used in this study: **(A)** A minimal model consisting of a spherical soma (blue), a long cylindrical dendrite (green) and a cylindrical axon (gray), **(C)** a detailed neuronal model (DM1) whose morphology was reconstructed from an L5b neocortical rat pyramidal neuron, and **(E)** another detailed neuronal model (DM2) whose morphology was based on that of a neuron from a *in vitro* 2D rat primary dissociated cortical culture. In each case, a 30 μm section of the proximal axon was defined as the axon initial segment (AIS; in red) and was assigned a high density of inactivating sodium and non-inactivating potassium channels. In the extracellular space around each neuron, we simulated a 30 x 30 HD-MEA with an electrode pitch of 17.5 μm. A 12 x 12 electrode block at the center (zoomed-in region) was chosen for a detailed analysis of extracellular “footprints”, i.e., the extracellular electrical potential landscape of a neuron across electrodes. **(B,D,F)** In each model, the AIS was the site of spike initiation. Intracellular voltages computed at various locations along the neuron revealed a clear temporal order with the initial peak at the AIS (red), followed by delayed peaks at the distal axon (gray), soma (blue) and the dendrite (green). Moreover, spike shapes at the AIS had a steeper slope during depolarization (**B**, lower inset). The somatic AP had a characteristic “kink” at its onset, which is observable as a break in the monotonous rise in the slope of the waveform [**(B)**, lower inset, zoomed in].

All neurons were simulated on a 30 x 30 grid of an HD-MEA with an electrode pitch of 17.5 μm (Figures 1A,C,E, orange). Individual electrodes were rectangular and of dimensions 9.3 × 5.45 μm. Out of this block, we used the central 12 x 12 electrodes for in-depth analysis of the neuron's extracellular “footprints,” i.e., representations of the extracellular electrical potential landscape measured across the array electrodes during action potential generation (Figures 1A,C,E middle panels). The electrode specifications were chosen to match that of the CMOS high-density microelectrode arrays (HD-MEAs) developed in our group (Ballini et al., 2014). The probe was designed and imported using the Python package, MEAutility^2^ (Buccino and Einevoll, 2021).

### 2.3. Simulation of neuronal activity

Action potentials were evoked in all models by injecting current steps (0.3–2 nA; duration, 20 ms) to the soma. Neuronal activity was simulated in Python using the Neuron-Python interface (Hines et al., 2009). The corresponding extracellular signals were simulated for the HD-MEA probe model using the LFPy package (Hagen et al., 2018). Simulations were run for 30 ms in time steps of 2^−5^s.

**Figure 2:**
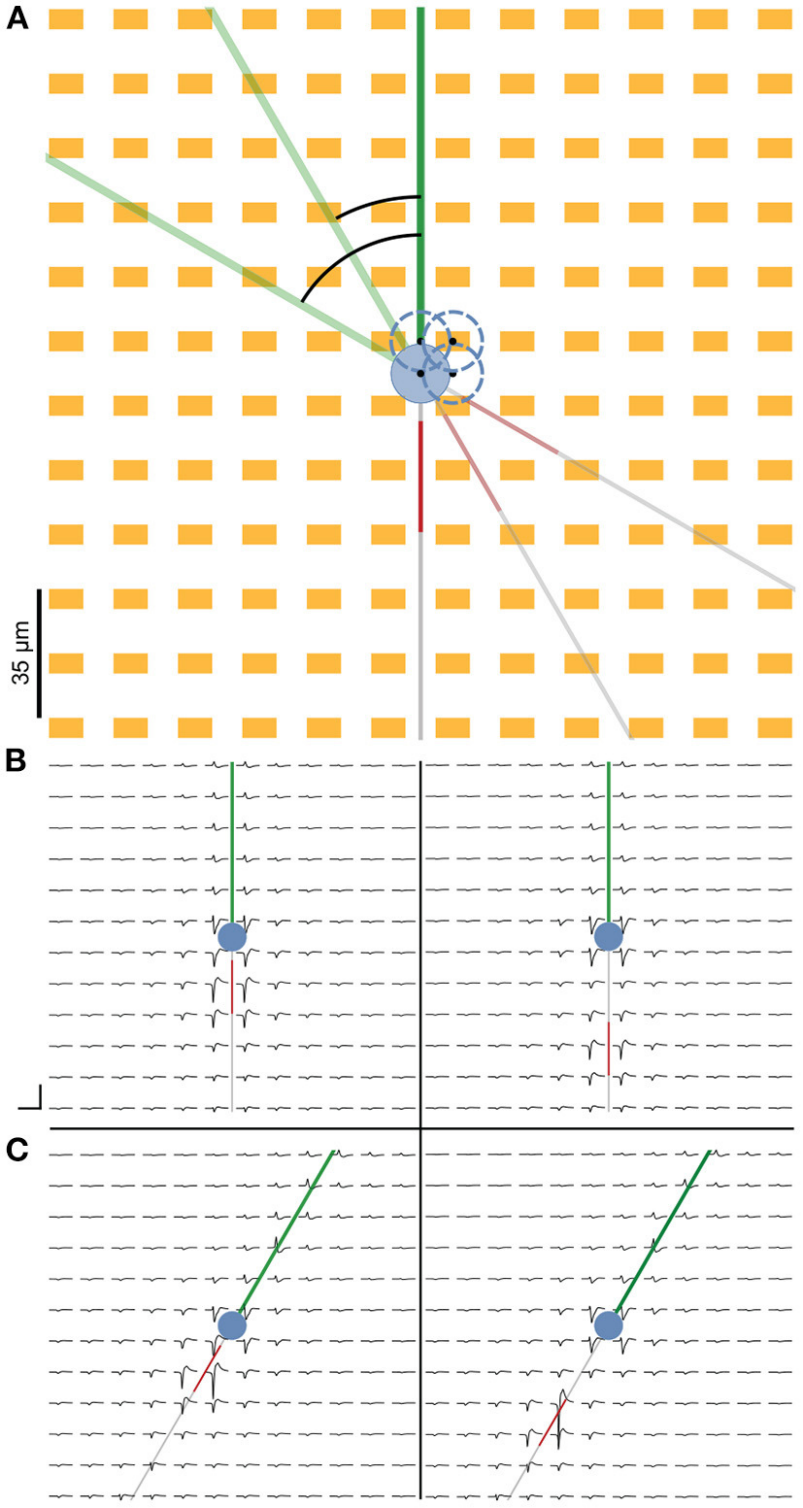
Representation of the cell on the simulated HD-MEA. **(A)** We simulated the neuron at four different somatic positions to account for extracellular footprint variability due to slight differences in the positions of the neural compartments relative to the electrodes. Neuron positions are indicated by the blue dashed circles, and the black dots represent the center of the soma. Additionally, the entire neuron was rotated 360 ° at 1° increments for each cell position to simulate the extracellular footprints pertaining to various possible neuronal orientations. **(B)** Simulated extracellular footprint of a cell with the baseline AIS starting 5 μm from the soma (left) and the farthest simulated AIS position being at 40 μm distance from the soma (right). **(C)** Extracellular footprint with the same soma position as in **(B)**, but with the neuron rotated clockwise by 30° relative to the baseline. Scale bar: 3 ms (horizontal) and 100 μV (vertical).

Extracellular footprint features are sensitive to the relative distances of neuronal compartments to the nearest electrodes. To average out this variability, we repeated the simulations corresponding to each AIS position with a slight soma positional translation. This jitter was restricted to within one electrode pitch owing to the periodic symmetry of the array. The four soma positions chosen were (0, 0), (8.75, 0), (0, 8.75), and (8.75, 8.75) where (0, 0) corresponds to the position at the mid-point of the square, formed by four adjacent electrodes (Figure 2A). All units were in μm and 8.75 was chosen because it was half the electrode pitch. The positional jitter also ensured that there were multiple slightly different electrical footprints in our training data, associated with a given AIS position and axonal orientation. This strategy adds to the robustness of the algorithm against natural footprint variability observed under experimental conditions as a consequence of small drifts/movements over the course of long recording durations (Supplementary Figure S6). Additionally, for each soma position, the entire neuron was rotated 360° around an axis centered at the soma. For the ball-and-stick model, we ran simulations in 1° increments. For each positional and rotational implementation, extracellular signals were generated for various AIS positions in 1 μm steps between 5 and 40 μm. This led to a family of 4 (positions) × 360 (angles) = 1,440 simulations for each of the 36 AIS positions. In the case of the detailed morphology models DM1 and DM2, to offset the added computational load, we chose 5° increments for the rotation and 2 μm steps of the AIS position, i.e., a family of 4 (positions) × 72 (angles) = 288 simulations for each of the 18 selected AIS positions. The neuron models, used in this study, and the scripts to generate the figures can be found on GitLab^3^.

### 2.4. Feature extraction

First, a short time snippet around the extracellular spike was cut out of each electrode of an extracellular footprint. We used a 96-sample (3 ms) cutout window for the ball-and-stick model, and a 177-sample (ca. 5.5 ms) cutout window for DM1 and DM2. The electrode that registered the earliest signal trough in each extracellular footprint was selected to compute the indices of the cutout window, and the same indices were used for each waveform of an extracellular footprint.

For each baseline extracellular footprint, amplitudes of the negative signal troughs were extracted based on the waveform cutouts. The top 26 electrodes, ranked by amplitudes, were selected. For cutouts from these channels, additional feature metrics were extracted including half-width, latency to earliest peak, peak-to-peak amplitude and slopes at half-maximum. The 26 selected electrode IDs were then sorted by latencies. This was done to preserve a rough sequential order of electrodes across extracellular footprints, with electrodes close to the AIS arranged early in the list.

A vector of feature changes as a result of AIS relocation (e.g., Δamplitudes, Δlatencies, etc.) was computed relative to the corresponding baseline extracellular footprint and between pairs of corresponding electrodes. For each feature change vector, a sham vector was computed by shuffling the feature vector before subtracting the baseline from it. A sham feature change vector would not correspond to a biophysically relevant signal and was labeled as an invalid AIS relocation. Shuffled footprints span a much larger possibility space, since it would also include physiologically implausible footprint configurations. Natural variability would, hence, be a proper subset of this space. Each vector was assigned a binary label depending on whether it was associated with a valid AIS relocation. Each valid feature-change vector was also labeled with the magnitude of the AIS relocation that it was associated with.

### 2.5. Detection of AIS plasticity and prediction of relocation magnitude

We first performed feature selection on the Δ feature vector using the neighborhood component analysis (NCA), a distance-based, supervised, non-parametric feature-selection technique (Goldberger et al., 2004; Yang et al., 2012). We computed the stochastic gradient-descent-based feature weights using a regularization parameter of λ=3 × 10^−5^. The resulting feature weights were thresholded at 10% of the maximum weight to eliminate features with low weights. Using the reduced set of features, we trained a random forest classifier to detect the status of AIS relocation (Breiman, 2001).

For predicting the magnitude of AIS relocation, we trained a regression model using the full feature vectors (without NCA). Wide neural networks with a single hidden layer of 100 RelU units were used for the task owing to their superior performance in hold-out data.

For both models, 60% percent of the simulated data were used for training, 20% for model validation, and the remaining 20% were held out to test the performance of the trained model. For the ball-and-stick model, hold-out validation was performed owing to the large size of the training set. For the detailed models, 5-fold cross-validation was performed.

Feature selection, machine learning, analyses, and data visualization were performed using custom scripts written in MATLAB^®^ (Version 9.11 (R2021b); MathWorks, Natick, MA).

### 2.6. Computation of kernel functions

AIS relocation systematically alters the extracellular electrical footprint around the neuron. Individual electrodes could be thought of as linear time-invariant (LTI) filters operating on baseline waveforms. An LTI system is completely characterized by its impulse response, and the output of each filter may be expressed as the convolution of the baseline waveform, *x*[*n*], with the impulse response, *h*[*n*] (eq.(1)). The impulse response is also known as the kernel function of the convolution sum.


(1) $$\begin{array}{l} y\left[n\right]=\left(h*x\right)\left[n\right]=\overset{+\infty }{\underset{m=-\infty }{\sum }}h\left[m\right]x\left[n-m\right] \end{array}$$

where *x*[*n*] is an individual waveform cut-out at an electrode of the baseline extracellular footprint and *y*[*n*], the corresponding transformed waveform after AIS relocation, both of length *N* samples, and *n*∈*W*. *h*[*n*] is the discrete impulse response with finite support (kernel function) corresponding to that electrode. Kernel functions were computed for pairs of extracellular footprints of the ball-and-stick model. Only *M*=26 electrodes per extracellular footprint, selected as described in Section 2.4, were used.

Since transformed waveforms are necessarily of finite length, kernels of finite length should be sufficient to describe these filters. The kernel length *L* reflects the complexity of the filter. Empirically, we determined that *L*=25 was sufficient to capture the waveform changes observed in our simulations.

To estimate kernel functions of length *L*, we recast the convolution operation as a matrix multiplication by defining a convolution matrix **X** such that:


y[n]=Xh[n]

**X** is then of size *N*×*L* and has a non-symmetric Toeplitz structure as shown below:


$$X=\left[\begin{array}{ccccc} x[0] & 0 & \cdots & \cdots & 0\\ x[1] & x[0] & 0 & \ldots & 0\\ \vdots & x[1] & x[0] & 0 & 0\\ \vdots & \vdots & \ddots & \ddots & \vdots \\ \vdots & \vdots & \ddots & \ddots & x[0]\\ \vdots & \vdots & \ddots & \ddots & x[1]\\ \vdots & \vdots & & & \vdots \\ x[N-1] & x[N-2] & \cdots & \cdots & x[N-L+1] \end{array}\right]$$

*h*[*n*] was then estimated as:


$$\hat{h}[n]=X^{-1}y[n]$$

In rare cases where **X** was ill-conditioned, we computed the Moore-Penrose pseudoinverse:


$$\hat{h}[n]={\left(X^{T}X+\delta I\right)}^{-1}X^{T}y[n]$$

where **X^T^** is the transpose of the convolution matrix, *I* is the identity matrix, and δ = 0.01 was added for numerical stability. The pseudoinverse was computed only for the ill-conditioned matrices to mitigate the computational load. For the well-conditioned cases, ĥ[*n*] was computed using Gaussian elimination without explicitly forming the inverse.

Using ĥ[*n*], and the baseline waveform for each electrode, a transformed waveform, ŷ[*n*], was estimated using the corresponding convolution matrix, **X**:


(2) $$\hat{y}[n]=X\hat{h}[n]$$

The normalized root-mean-squared (RMS) error, *E* between actual and estimated traces for a single electrode, given a kernel function, was computed as:


(3) $$E|\hat{h}[n]=\frac{1}{\left({\hat{y}}_{max}-{\hat{y}}_{min}\right)}\sqrt{\frac{1}{N}\sum_{n=0}^{N-1}{\left(\hat{y}[n]-y[n]\right)}^{2}}$$

where ŷ_*max*_ (resp. ŷ_*min*_) is the maximum (resp. minimum) value of the estimated waveform.

Using the entire set of simulations, a kernel library was compiled as a discrete functional, **H**[*m, d*_*ais*_, θ], parameterized by the electrode index, *m*, AIS position, *d*_*ais*_, and rotation of the neuron, θ. That is, for each combination of the three parameters, **H**[·] was a mapping between the parameter space and impulse response functions pertaining to the extracellular footprint transformation at electrode *m*, corresponding to an AIS position, *d*_*ais*_, of a neuron model that was rotated by an angle θ. The waveforms at *d*_*ais*_= 5 μm were used as the baseline condition for all transformations.

When estimating waveforms at the *m*^*th*^ electrode for a detailed morphology model, we could not directly use a kernel function corresponding to electrode *m* of the extracellular footprint. This was because the kernel functions were computed only based on the ball-and-stick morphology and there was no electrode-by-electrode morphological correspondence between the models. Ideally, an electrode in the ball-and-stick model, m^*^ that matches the morphological compartment close to a given electrode *m* in the detailed morphology model should instead be chosen. Mapping such a selection manually would be tedious and would presume full morphological knowledge of the neuron being studied. Although, in our case, full morphological details were available, this may generally not be the case in a high-throughput experimental setting. Hence, instead of relying on the available morphology, we estimated m^*^ on the fly for each electrode *m* in extracellular footprints of the detailed morphology models, for a given AIS position, *d*_*ais*_, by searching in the kernel library over all angles for the electrode corresponding to the lowest prediction error:


(4) $$m^{*}=\underset{m}{\arg\min}\;E|H[m,d_{ais},\cdot ]$$

The mean normalized RMS error given an AIS position and orientation, $\bar{E}|d_{ais},\theta$, was then computed by averaging the normalized RMS error over all the *M* selected electrodes in the extracellular footprint:


(5) $$\bar{E}|d_{ais},\theta =\frac{1}{M}\sum_{m=1}^{M}E|H[m^{*},d_{ais},\theta ]$$

## 3. Results

### 3.1. The AIS is the site of action-potential initiation in our models

We used three distinct neuron models in this study: a minimal ball-and-stick model, and two detailed multi-compartment models (detailed morphology-1 and 2, abbreviated as DM1 and DM2). The minimal morphology model consisted of a circular soma, a cylindrical dendrite and a thin cylindrical axon (Figure 1A). A section of the proximal axon was defined as the axon initial segment (AIS) and was endowed with a highly uniform density of inactivating sodium and non-inactivating potassium channels (Figure 1A, red).

The two detailed multi-compartment models were based on reconstructed morphologies. The morphology of DM1 was reconstructed from an L5b neocortical rat pyramidal neuron. The morphology of DM2 was reconstructed from a neuron in a primary rat dissociated cortical culture *in vitro* at DIV 22 (see Section 2). In all three models, we inserted a 30 μm long AIS in the proximal axon. The AIS position, parameterized by the distance between the soma and the proximal end of the AIS, was systematically varied between 5 and 40 μm.

To verify the functional role of the modeled AIS in action-potential initiation and as a major contributor to extracellular electric fields, we injected a current pulse at the soma and studied the triggered action potential waveform intracellularly at the following sub-cellular compartments: within the first 70 μm of the dendrite (green), soma (blue), the midpoint of the AIS (red), and within the first 70 μm of the axon. In each of our models, the earliest electrical signal peak—hence the action potential initiation—was observed at the AIS, whereas the somatic AP occurred with a delay (Figures 1B,D,F). Our observation is in agreement with experimental observations of AP initiation (Stuart et al., 1997). Consistent with previous reports, we also observed a characteristic “kink” at the onset of the somatic AP, which is thought to be the result of resistive coupling between the AIS and the soma (Telenczuk et al., 2017). The kink is clearly visible in the blue dashed line Figure 1B (lower inset), and can be seen as a transient break in the monotonous rise in the time derivative of the somatic voltage trace.

Moreover, intracellular voltage traces during the depolarizing phase were steeper at the AIS than at the soma (Figure 1B dashed lines; Supplementary Figure S2). In the ball-and-stick model, the peak value of the voltage derivative at the AIS (1,430 mV/ms) was more than twice that of the somatic trace (557 mV/ms). This was also the case for both detailed morphology models (>6- and >5-fold increase for DM1 and DM2 respectively; Supplementary Figure S2). In all three models, the AIS was thus the site of larger trans-membrane current intensities and was—hence—likely a dominant contributor to extracellular potentials around the neuron (Bakkum et al., 2019).

### 3.2. AIS relocation was associated with systematic changes in extracellular footprints

Extracellular potentials at individual electrodes could vary depending on the distance and orientation of individual neurites relative to the electrodes. To capture this variability, we simulated extracellular potentials at various soma positions and neurite orientations (Figure 2A). For each positional and rotational neuron implementation, extracellular signals were generated for various AIS positions (see Section 2).

Extracellular electric potentials around the neuron were simulated with the LFPy package (Hagen et al., 2018). Signals were computed at the locations specified by the electrode design and consisted of 30 ms long traces of activity concurrent to the injection of a current step at the soma. The site of the earliest negative amplitude peak was identified, and indices were computed around it that corresponded to a ca. 3 ms cut-out. Array-wide snippets were cut out using these indices and visualized as the extracellular footprint.

Figures 2B,C illustrates the extracellular footprint for the ball-and-stick neuron corresponding to various AIS positions and neuronal orientations. In general, the AIS was the site of the earliest occurrence of a electrical signal trough. For the ball-and-stick model, it was also the site where the largest negative signal amplitude occurred. The dominant sinks, corresponding to the troughs in the waveform, can be seen to move in tandem with distal shifts of the AIS position.

**Figure 3:**
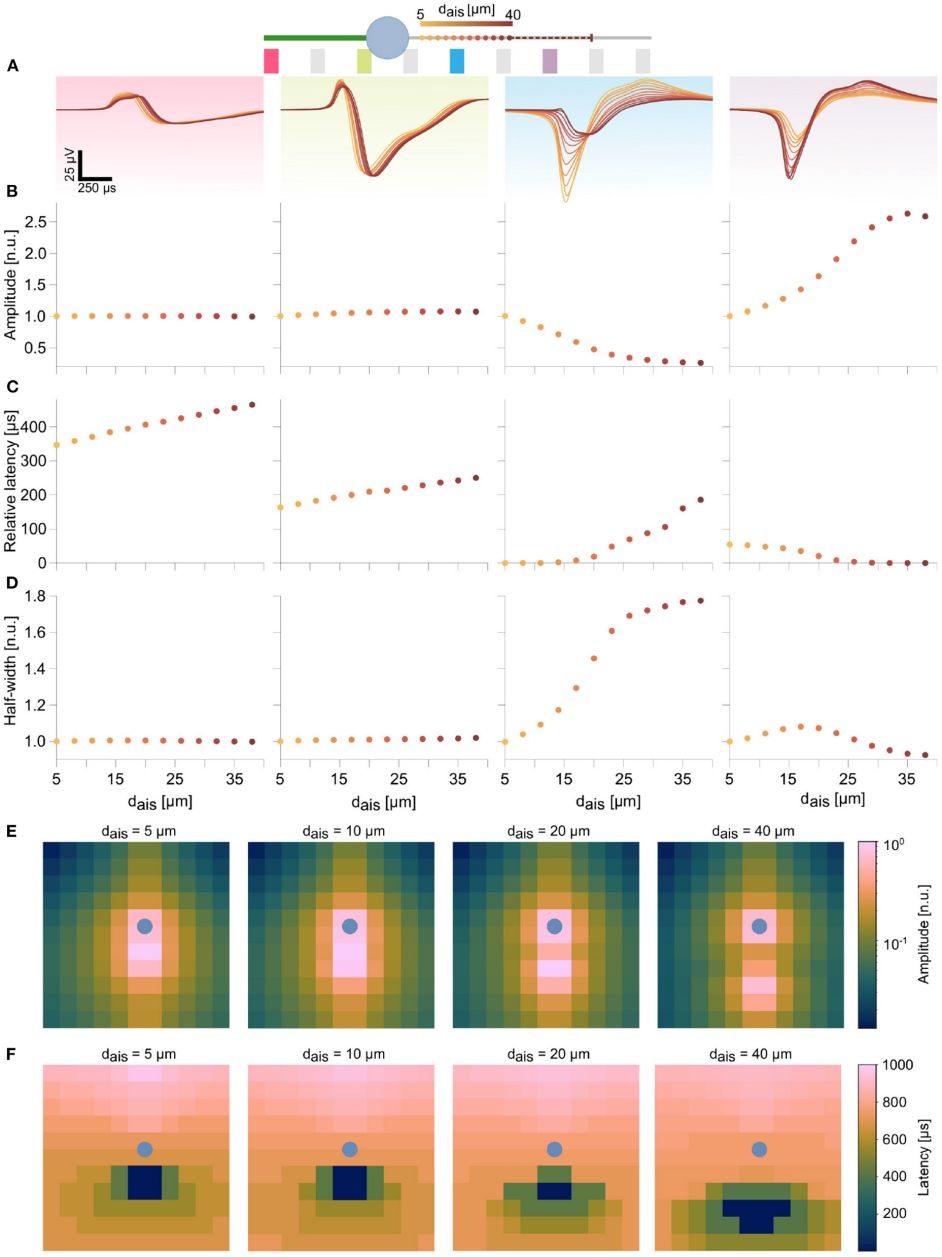
Features in the extracellular footprint of the ball-and-stick model exhibited systematic changes in dependence of AIS relocation. **(A)** Waveforms at selected electrodes (pink, green, blue, and purple) along the neuron are shown for various AIS locations. Proximal AIS locations are indicated as dots whose colors represent distances to the soma (see text footnote^4^). The dashed line represents the AIS length, when starting from the final position. **(B)** Amplitudes along the dendrite (pink) and close to the soma (green) did not change with changes in AIS positions. AIS relocation away from an electrode resulted in amplitude losses (blue), while the reverse was true when the AIS moved closer to an electrode (purple). **(C)** AIS relocation away from the soma resulted in delayed peaks at the dendrite and soma, but earlier peaks (decreasing latencies) were observed downstream of the AIS. **(D)** Proximity of the electrode to the AIS was generally associated with sharper spike shapes (blue, purple electrodes). Amplitudes and half-widths were normalized to the respective baseline values (*d*_*ais*_=5 μm), while latencies were computed relative to the earliest detected signal peak for each AIS position (see Supplementary Figure S3 for non-normalized values). The log-scaled amplitude **(E)** and latency **(F)** spatial maps over a 12 x 12 electrode block at various AIS positions reflect the trends described in **(B,C)** over multiple electrodes. The respective soma position is indicated by the blue dot.

We analyzed specific waveform features at individual electrodes during AIS relocation. For the ball-and-stick model, 4 electrodes along the soma and the neurites were selected to illustrate these features (Figure 3A). AIS positions are indicated as dots along the axon, and the dot color (light to dark) represents increasing distances to the soma^4^. For each of the selected electrodes, we studied the alterations of the signal trough amplitudes, latencies relative to the earliest trough, and waveform widths at half amplitudes (half-widths). Amplitudes and half-widths were normalized to values corresponding to the baseline position of the AIS (i.e., 5 μm).

In peridendritic (pink) and perisomatic (green) electrodes, signal amplitudes and half-widths were unaffected by AIS relocation, though latencies increased with AIS distance, which reflected the delay of the generated AP to propagate back into these compartments (Figures 3B–D and Supplementary Figure S3). In contrast, the blue electrode, close to the baseline AIS position, recorded a substantial drop in signal amplitude along with a broadening of the waveform. Though initially it was the earliest peak position (*latency*=0), latencies were found to increase, as the AIS moved distally (Figure 3C). Conversely, at the purple electrode, located in the movement direction of the relocating AIS recorded progressively increasing signal amplitudes while latencies steadily decreased to zero. The initial tendency for the waveform to widen was quickly reversed, as the AIS moved closer to the electrode (Figures 3B–D). In the meantime, only modest changes in these signal features were observed further down the axon (not shown). The spatial maps of amplitudes and latencies, computed at 4 different AIS positions over 144 electrodes in the 12 x 12 block summarize these trends (Figures 3E,F). As the AIS relocated distally, the latency map changed accordingly. A clear spatial separation of the dominant sinks into two groups around the AIS and around the soma was observed in the amplitude map (Figure 3E right-most panel). The soma position is marked as a blue dot in these maps.

**Figure 4:**
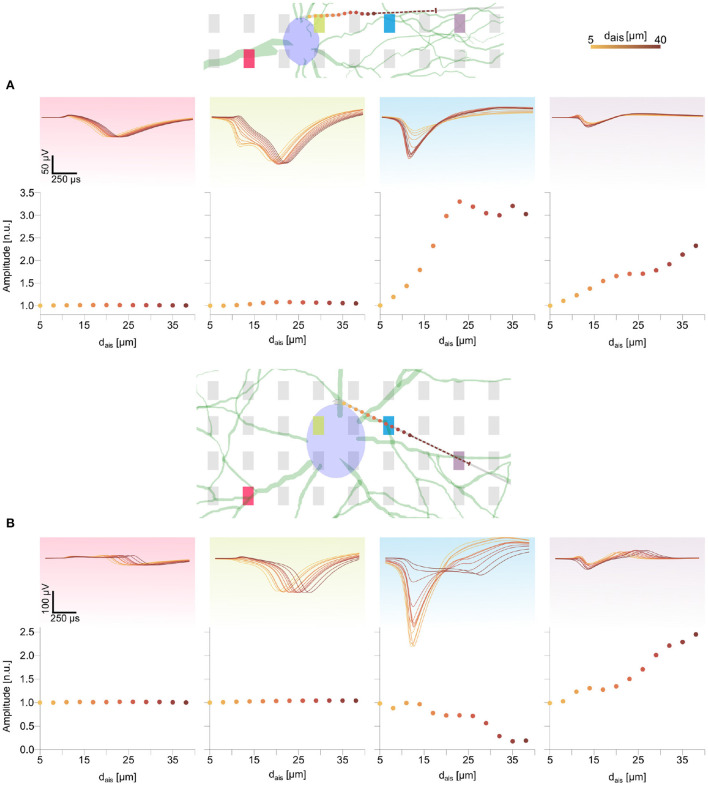
Changes in spike waveform features for the two detailed morphology models were qualitatively similar to those observed in the ball-and-stick model (Figure 3). The AIS locations are marked as colored dots along the axon (see text footnote^4^). The dashed line illustrates the 30 μm long AIS, starting from the last position. Normalized extracellular amplitudes are shown in the bottom rows of **(A,B)**. Movement of the AIS away from an electrode along the axon led to extracellular amplitude attenuation [e.g., blue electrode and panel in **(B)**], whereas amplitudes increased for electrodes in the direction of the AIS relocation [e.g., purple electrode and panel in **(B)**].

Feature changes of a similar nature were observed for the DM1 and DM2 models (Figures 4A,B and Supplementary Figure S4). The waveform features on peridendritic (pink) and perisomatic (green) electrodes generally were preserved, while electrodes in the direction of the AIS relocation recorded increasing amplitudes and sharper spike shapes as observed on the blue electrode in DM1 (Figure 4A) and the purple electrode in DM2 (Figure 4B and Supplementary Figure S5).

### 3.3. Feature changes in the extracellular footprints are reliable predictors of AIS plasticity

Next, we investigated if these systematic trends in the spatio-temporal characteristics of the extracellular footprints could be fruitfully exploited as reliable indicators of AIS plasticity.

To do so, we spawned a family of simulations for each neuron model, as described in earlier sections. The simulated signals were used to systematically construct a large possibility space of extracellular footprint feature changes that could or could not (i.e., sham cases) be associated with AIS relocation. See Section 2 for a description of the generation of these feature vectors. Each extracellular footprint pair was assigned a binary label to indicate whether it involved AIS-relocation-mediated changes.

Feature dimensions were reduced by pre-selecting the top 26 electrodes according to signal amplitude within the baseline extracellular footprint. These electrodes were then sorted by latency from the earliest to the latest occurrence of the signal trough. Feature differences in corresponding electrodes were stored in a vector. In a second step, we computed feature weights using the neighborhood components analysis (NCA), a distance-based, supervised, non-parametric feature-weighting technique. The weights reflected the degree to which each feature was relevant to detecting AIS relocation. We then selected the most important features by thresholding feature weights and used these to train our classifiers.

**Figure 5:**
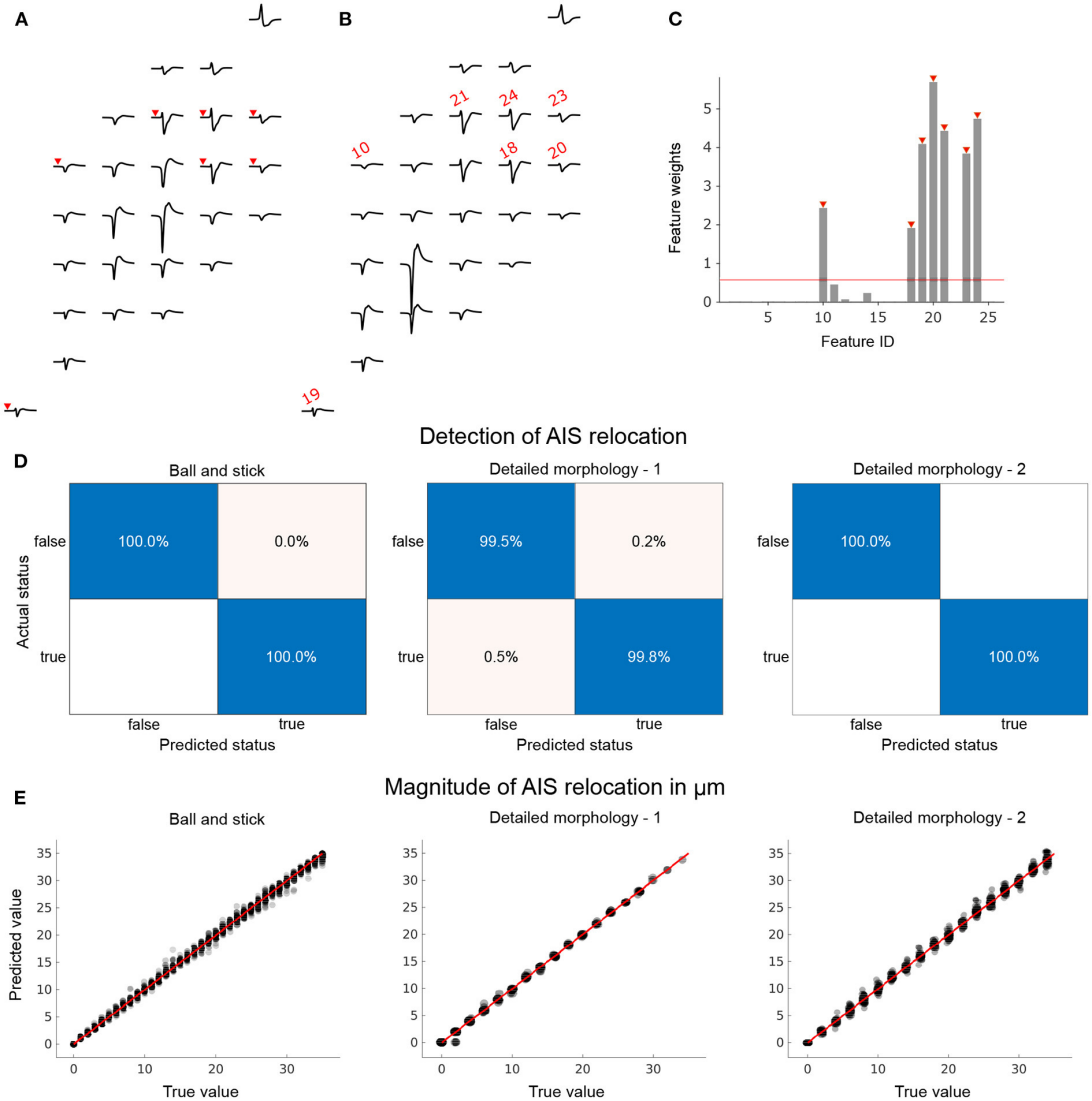
Models to reliably detect AIS relocation and predict its magnitude can be trained by using a family of extracellular activity simulations initialized by the respective baseline morphologies. We pre-selected a subset of electrodes from the extracellular footprints to constrain the feature space for statistical learning. 26 electrodes with the highest amplitudes in baseline configuration (initial AIS position) were pre-selected. Amplitude changes at these electrodes were computed between baseline **(A)** and relocated **(B)** extracellular footprints. The footprints shown were based on a ball-and-stick model with a 30° rotation with AIS positions at 5 μm (baseline) and 40 μm (relocated). **(C)** For the detection of AIS relocation, an additional feature-selection step using neighborhood components analysis (NCA) was performed. Feature IDs marked in red (corresponding electrodes in **(A,B)** are marked in red with arrowheads or the feature IDs) were selected to train the classifier (relocation status detector). **(D)** The trained classifiers performed very well on the held-out test data sets for each of the three neuron models. True positive rates were high and false positives were negligible or non-existent (blank fields). **(E)** Changes in amplitude were used to train a regression model to predict the magnitude of AIS relocation. Trained models were tested using held-out data sets and showed remarkable predictive power for each neuron model.

The procedure is illustrated for a pair of ball-and-stick extracellular footprints that were associated with AIS relocation (Figure 5). Δ amplitudes were used to construct the feature vector in this example. Feature weights computed with NCA were thresholded to select the most relevant features (7 in this case; Figure 5C). The selected feature IDs are mapped on to the example extracellular footprints (red) in Figures 5A,B. Notably, electrodes with prominent changes in the footprint, shown in Figures 5A,B, were not selected, presumably because they were not reliable indicators of AIS plasticity. The pooled training data contained footprints from various AIS locations. Consequently, the positions and magnitudes of these electrodes featuring large changes would be highly variable.

The feature vectors were used to train classifiers for each morphology. Their performances were tested using hold-out data sets that were not used to train or validate the models. In each case, and independent of morphological complexity, the trained model was able to detect with near-perfect accuracy whether or not the given pair of extracellular footprints were associated with AIS relocation (Figure 5D).

We then asked if the same features could be used to infer the magnitude of relocation that was involved in the extracellular footprint transformation. Only extracellular footprint pairs associated with a valid AIS relocation were considered for this task. To this end we trained regression models using Δ amplitude features. Since we did not observe significant differences in feature weights computed by NCA, we used all available features to train the regression model. The performance of the models was evaluated using held-out data: it was possible to make reliable predictions based on pair-wise features extracted from the extracellular footprints around the respective neurons. The magnitude of AIS relocation associated with each feature vector in the hold-out data set (true value), predicted values based on the trained model, and the null-error line (red) are shown in Figure 5E.

### 3.4. Extracellular footprint transformations captured by kernel functions can predict AIS plasticity even in the absence of morphological information

We showed that by instantiating a family of simulations using the baseline morphology, it was possible to reliably detect and predict the magnitude of AIS relocation using waveform feature changes observed in simulated extracellular potentials.

However, to apply this method to a potential HD-MEA based experimental setting, involving hundreds to thousands of neurons, it would be necessary to collect morphological information pertaining to each neuron and run customized simulations to generate the simulated feature change vectors. This could be a computationally expensive procedure. Hence, we explored a scalable alternative where running simulations customized by morphology for each unit would be unnecessary.

We attempted to capture extracellular footprint transformations, specifically those associated with AIS plasticity, in a mathematical description that could then be efficiently generalized over neuronal morphologies. To this end, we abstracted the phenomenon of AIS relocation as a linear transformation that could then be completely characterized by its impulse response. Ideally, kernel functions derived from a simple ball-and-stick model should be translatable and transferable across morphologies.

**Figure 6:**
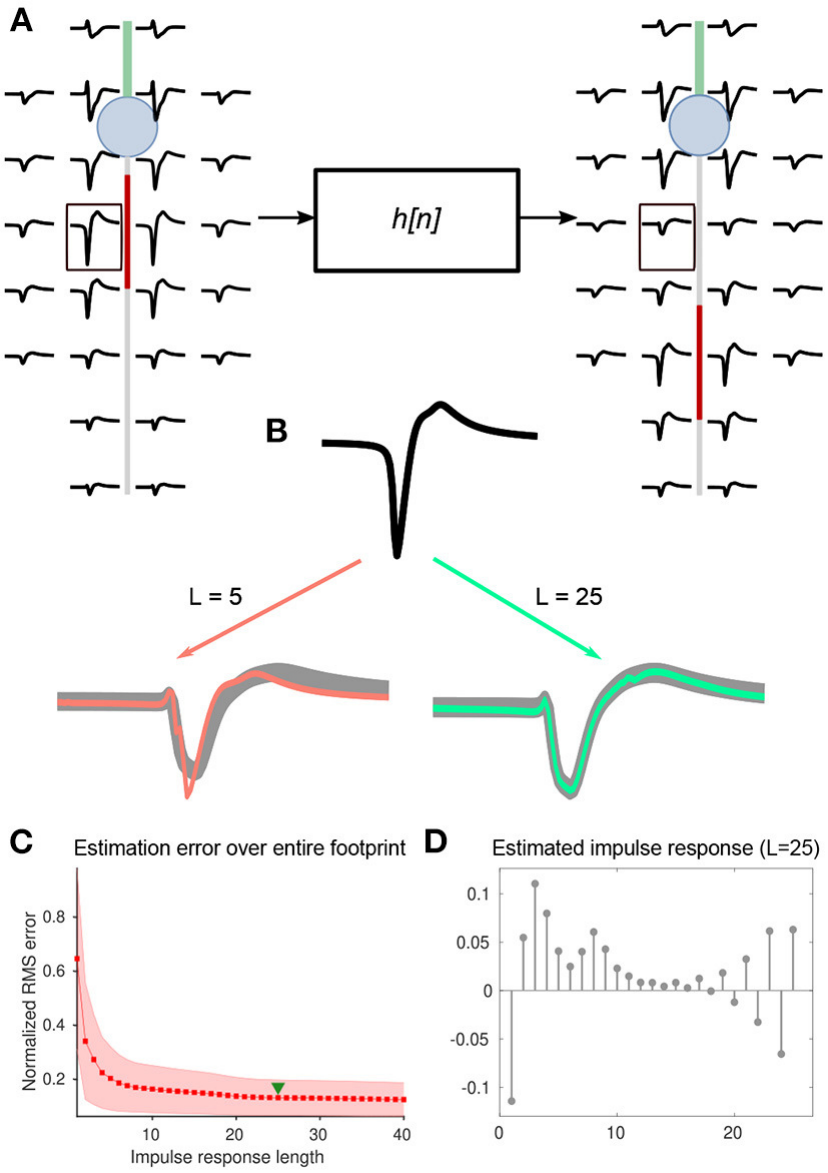
**(A)** Changes in each pair of extracellular footprints, associated with AIS relocation, were modeled as a set of *M* linear transformations, each associated with an impulse response, *h*_*m*_[*n*], where *m*∈{1, ⋯, *M*} denotes the electrode number, and *M* is the number of electrodes in the extracellular footprint. Electrode-wise impulse responses of length *L*=25 were estimated using the inverse of the convolution matrix (see methods). **(B)** Illustration of the impulse response estimation for a single electrode marked (black box) in **(A)**. The baseline and transformed waveforms are shown in black and gray respectively. Overlays of transformed waveforms, estimated using the impulse response of lengths L = 5 and 25, are shown in orange and green, respectively. **(C)** The RMS error normalized and averaged across electrodes decreased steadily with increasing impulse-response lengths. We used a length of 25 for further analyses (green triangle). **(D)** A sample impulse response (L = 25), computed for the waveform transformation at the electrode shown in **(B)**.

To test this idea, we estimated discrete kernels for each electrode and AIS position—always on a pairwise basis—relative to baseline extracellular footprints (i.e., AIS position at 5 μm; Figure 6A). In general, a longer kernel yielded a better estimate of the transformation (Figure 6B). By assessing the mean normalized root-mean-squared error between actual and estimated waveforms, we chose to use kernels of length 25 for further analyses (Figures 6B,C). A library of estimated kernels was stored, which was parameterized by axonal orientation, and the magnitude of AIS relocation that was used for the simulation (Figure 6D).

We tested the kernel library that has been computed using the ball-and-stick model on the two detailed neuronal models, DM1 and DM2. We started with a pair of extracellular footprints of the detailed morphology model: a baseline extracellular footprint and one after AIS relocation of unknown magnitude.

**Figure 7:**
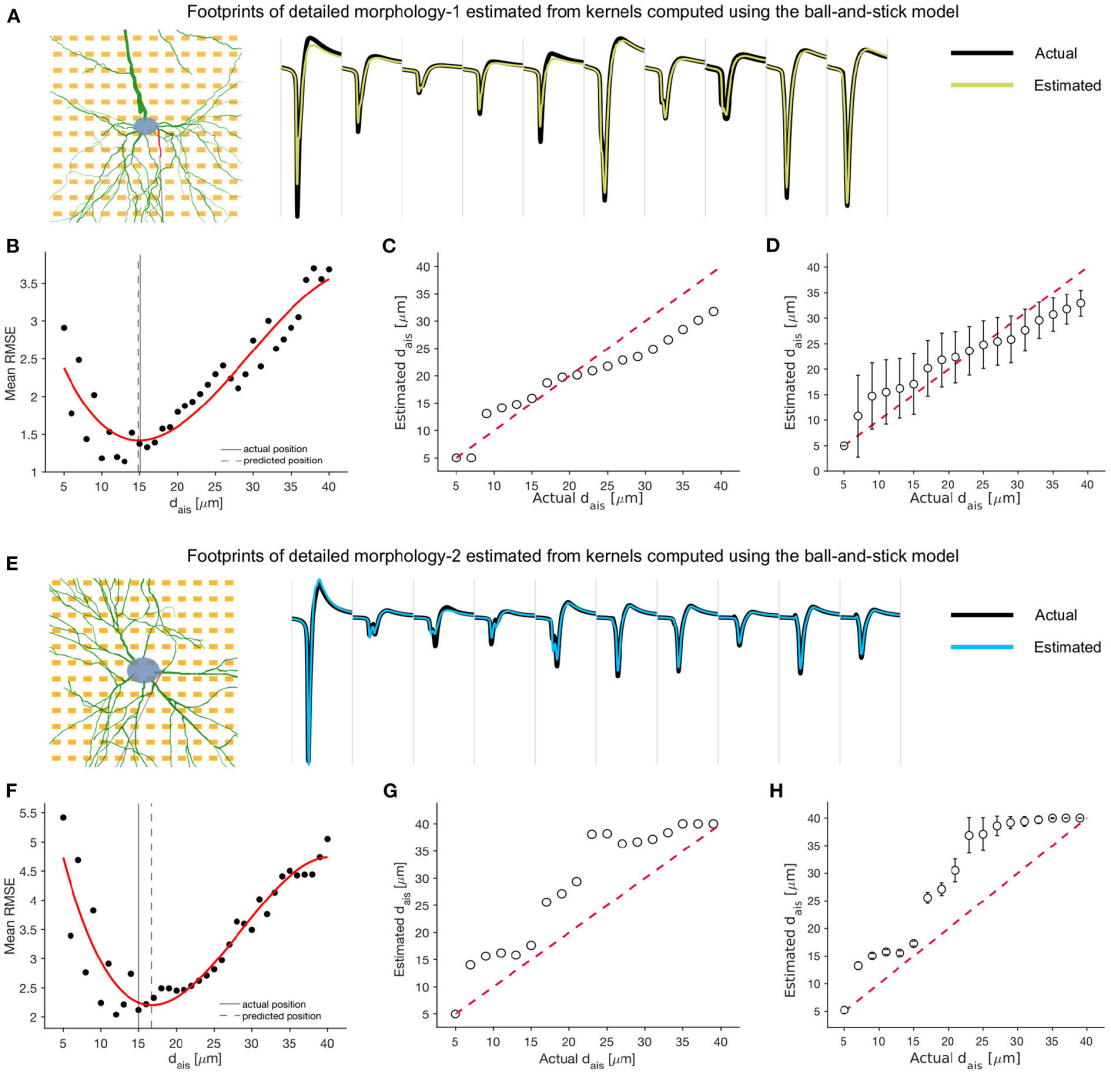
Kernel functions, derived from the minimal ball-and-stick model, may be used to detect and predict AIS relocation relative to baseline extracellular footprints, even when the specific baseline morphology is unavailable. **(A,E)** Waveforms from 10 selected channels of the extracellular footprint of the detailed morphology models corresponding to an AIS position of 15 μm (black) and traces estimated by the optimal kernel functions (yellow: DM1, blue: DM2). **(B,F)** The mean RMS error between the actual and estimated extracellular footprint averaged over electrodes and computed using kernel functions corresponding to various AIS positions. The error is lowest around the actual AIS position (15 μm). A cubic polynomial was fitted (red) to the errors, and the putative AIS position was estimated as the one corresponding to the minimum of this fitted function. The traces in [**(A)**, yellow] and [**(E)**, blue] were estimated using this optimal kernel. **(C,G)** Comparison of actual and estimated AIS positions for a single orientation (θ=0°). Each estimate was the mean over four jittered soma positions. **(D,H)** The mean and standard deviation of each estimate, averaged over all rotations of the neuron model.

Next, we forward-estimated the transformed extracellular footprint using each set of kernels, stored in our library, and estimated the mean RMS error relative to the actual transformed extracellular footprint (see Equation 3–5; Figures 7B,F). We fitted a cubic polynomial to the computed errors and found that they were convex over the tested interval, with a clear global minimum, provided the extracellular footprint transformation was associated with a ‘valid' AIS relocation. The estimates, made with the best fitting kernels, were overlaid with selected electrodes from the actual extracellular footprints, and we observed a high degree of agreement (Figures 7A,E).

We found that the AIS position corresponding to the set of best kernels could be used as a rough estimate of the AIS relocation. For the case of no rotation and a single soma position, we tested various extracellular footprints corresponding to AIS positions between 5 and 40 μm. The kernel-based best estimates aligned roughly along the null-error diagonal (red dashed) for both DM1 (Figure 7C) and DM2 (Figure 7G). Similar estimates were made for various axonal orientations between 0° and 360°, and the mean (std) of the estimated AIS positions were reported for each model (Figures 7D,H). Overall, the kernel-based technique could be a promising approach for efficient extracellular data-based high-throughput detection and prediction of AIS relocation.

## 4. Discussion

We established an analysis framework to reliably detect and track structural plasticity of the axon initial segment (AIS) in simulated extracellular electrical readouts of HD-MEAs. We proposed two computationally assisted methods with the goal to enhance the throughput of experimental studies of AIS plasticity, especially in *in vitro* models: i) a method based on implementing a family of replicate simulations and ii) a method based on linear systems theory.

The AIS was recently reported to exhibit structural modifications in response to chronic alterations in neuronal activity (Grubb and Burrone, 2010). These structural modifications also appear to help restore initial excitability levels of neurons and are, thus, thought to play a stabilizing role (Lezmy et al., 2020). The AIS is a very versatile and complex microdomain, and many aspects of its plasticity and functional role are still poorly understood. One of the reasons is the large variety of the possible structural alterations—distal/proximal shifts, shortening and lengthening have all been reported (Grubb and Burrone, 2010; Kuba et al., 2010; Evans et al., 2013; Chand et al., 2015; Lezmy et al., 2017). Moreover, a layer of complex multi-factor dependencies seems to influence the phenomenon and its functional role. These include, for example, neuron type, soma size, and the baseline AIS geometry.

Studies of AIS plasticity to date predominantly relied on comparative assays of snapshots between control and perturbed groups and have important limitations (Dumitrescu et al., 2016). Since the averages of different sets of neurons were studied in each group, it is difficult to control for sources of variability (especially unique morphological properties). This issue could be resolved by tracking the same neuron over a long duration, from baseline state to a state with a modified AIS. Simultaneous access to activity in the network would be desirable to attempt to tease apart the contributions of individual neuronal modifications to the network activity.

A potential solution could be to live-image structural AIS plasticity. Dumitrescu et al. (2016) recently proposed that a fluorescently-tagged sodium channel motif—YFP-Na_*V*_II-III—may be suitable for time-lapse imaging of activity-dependent AIS plasticity, although certain aspects (like the delayed response of the probe relative to endogenous AIS proteins) are not yet fully understood. The imaging approach has the advantage that it could also reveal the detailed neuronal morphology when used in combination with high-resolution microscopy, however, it does not offer access to neuronal activity. Nevertheless, it could be combined with simultaneous functional imaging, for example, using calcium probes or genetically encoded voltage indicators (Abdelfattah et al., 2019). However, an all-optical approach may be technically challenging. Moreover, it is important to consider that excitation-light exposure can have significant photobleaching and phototoxicity effects that may compromise reliability and reproducibility of the obtained data, especially when imaging is done at high spatial resolution (Icha et al., 2017). Low experimental throughput, poor temporal resolution (for calcium imaging), and inadequate signal-to-noise ratio (for voltage imaging) are further disadvantages of an optical approach.

The AIS, by virtue of its unique molecular architecture, is the location of highest electrical excitability in the neuron and is the dominant contributor to extracellular electric fields (Radivojevic et al., 2016; Bakkum et al., 2019). For this reason, it is amenable to investigations using high-resolution extracellular electrophysiology. HD-MEAs are capable of providing stable, label-free, long-term readout of electrical activity at high spatio-temporal resolution (see Supplementary Figure S6; Ballini et al., 2014). What is missing is an analysis framework to reliably identify and capture specific activity signatures that can be uniquely mapped to underlying structural changes at subcellular scale.

In this study, we present a first attempt at tackling this challenge. We used computational models to characterize extracellular footprint changes in response to specific microstructural modifications. In order to generalize the results, we studied changes in pairs of extracellular footprints. Such synthetic data could be used to train machine-learning models (method 1) or be used to extract kernel functions to predict waveform transformations associated with AIS plasticity (method 2).

Method 1 relies on baseline morphological information to spawn replicate simulations. While this method could involve substantial computational costs, this is not necessarily a disadvantage. AIS plasticity and its functional consequences have been tied to various morphological parameters like axon diameter, soma size, etc., the reliable inference of which is beyond the scope of a pure extracellular-electrophysiology-based approach. Therefore, a determination of initial neuron morphology will add significant value to the analysis.

Our second method has the advantage that it is fast, has low computational overhead, and relies on a library of already computed kernel functions that may—in principle—be progressively improved, as more data are gathered. Further, the search for an optimal kernel may be rendered more efficient: currently, a kernel search over all possible angles is performed, whereas, if the actual orientation of the proximal axon could be estimated, the kernel search space can be restricted accordingly. Such an estimation could be possible based only on the extracellular footprint. For example, a regression model may be trained to predict the orientation of the proximal axon as demonstrated for the ball-and-stick model (Supplementary Figure S1).

To develop the algorithm and illustrate its effectiveness, we focused on distal relocation of the AIS. However, the approach itself is flexible and can readily be extended to other AIS plasticity phenomena like changes in length, diameter, or relative densities of the AIS protein machinery, and diverse baseline AIS morphologies—including those localized at axon-carrying dendrites (Höfflin et al., 2017). For each case, we would need to run a new set of simulations and train or compute the corresponding models. Aberrant phenomena like neurons with multiple functional AISs have also been reported *in vitro*, though it is unclear if and how structural plasticity is expressed in such neurons (Guo et al., 2017). Hence, such cases will be challenging to reliably model using our approach.

A constant AIS length of 30 μm was used throughout this study. The AIS length is, in general, a very important parameter and needs to be systematically explored in future studies. The minimal HD-MEA electrode pitch necessary to precisely discriminate an incremental change of the AIS is an interesting constraint that needs to be explored.

The dominant contributor to the spatial variability in the extracellular footprint would be the morpho-geometric properties of a given neuron. This issue could be addressed by reconstructing experimentally observed neuronal morphologies, as we do in this study. Another possible source of extracellular footprint variability could be the diversity in the electrophysiological properties of primary neurons *in vitro*. Modeling this variability could potentially add to the robustness of our approach.

Experimental studies are necessary to thoroughly validate the premise of our study. Carefully curated and labeled ground-truth data sets, for example, combined live-imaging and HD-MEA readouts, are essential to benchmark the recall and accuracy of our approach. Additionally, various parameters may need tuning to suit experimental data. For example, the choice of 26 electrodes to featurize each extracellular footprint was a heuristic guess and may need to be adjusted depending on experimentally available extracellular footprints. Further, electrode pre-selection based on feature thresholding (e.g., amplitude, latency) could be improved using sophisticated approaches. The recently published automated axon tracking package could, e.g., be used to estimate the orientation and bearing of the proximal axon, and electrodes neighboring the estimated trajectories could be used to better engineer feature vectors (Buccino et al., 2022b).

In conclusion, extracellular microelectrode array data constitute a rich and complex mixture of contributions from multiple neurons and neuronal compartments. Harvesting reliable information that can be mapped back to the underlying biological sources has been an outstanding computational challenge. A variety of methods, ranging from simple threshold-based detection of multi-unit spiking to more sophisticated unsupervised algorithms (e.g., spike sorting), has been proposed and progressively improved to add value to extracellular electrophysiology. Alongside, technological advances have enabled significant strides in spatial and temporal resolution of recording techniques. In addition, better computational schemes need to be developed to augment the interpretational value of extracellular electrophysiological data and to reliably elucidate the underlying biological phenomena. Our study is a contribution in this direction.

## Data availability statement

The raw data supporting the conclusions of this article will be made available by the authors, without undue reservation. The neuron models used in this study, and the scripts to reproduce the results are available at: https://git.bsse.ethz.ch/srkumar/tracking-ais-plasticity.git.

## Ethics statement

The animal study was reviewed and approved by Basel-Stadt Veterinary Office.

## Author contributions

SK, VE, and AB contributed to conception and design of the study. SK, AB, and TG contributed to the coding. SK and TG performed the simulations, the analysis, and wrote the first draft of the manuscript. AB, XX, JB, and TG performed experiments to reconstruct the neuron morphology and wrote sections of the manuscript. Project supervision was provided by AH. All authors contributed to manuscript revision, read, and approved the submitted version.

## Funding

This work was supported by the ERC Advanced Grant 694829 neuroXscales, the SNSF under contract 205320_188910/1, and the ETH Zurich Postdoctoral Fellowship 19-2 FEL-17 (AB). Open access funding provided by ETH Zurich.

## Conflict of interest

The authors declare that the research was conducted in the absence of any commercial or financial relationships that could be construed as a potential conflict of interest.

## Publisher's note

All claims expressed in this article are solely those of the authors and do not necessarily represent those of their affiliated organizations, or those of the publisher, the editors and the reviewers. Any product that may be evaluated in this article, or claim that may be made by its manufacturer, is not guaranteed or endorsed by the publisher.

## Footnotes

^1^Huygens Professional: http://svi.nl.

^2^MEAutility: https://meautility.readthedocs.io/.

^3^GitLab: https://git.bsse.ethz.ch/srkumar/tracking-ais-plasticity.git.

^4^All colormaps from Crameri et al. (2020).
