# Supplementary material for: Tracking axon initial segment plasticity using high-density microelectrode arrays: A computational study
Source: Front Neuroinform. 2022 Oct 3;16:957255. doi: 10.3389/fninf.2022.957255 (PMC7613690; doi:10.3389/fninf.2022.957255)
Supplement: Supplementary file 1 [file Data_Sheet_1.pdf]

Supplementary Material

1 SUPPLEMENTARY FIGURES

1.1 Figures

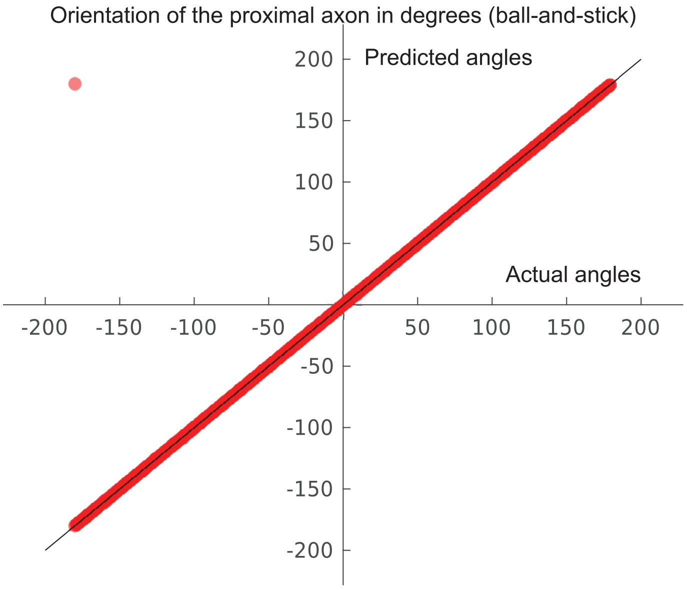

Figure S1. The orientation of the proximal axon can be reliably predicted based on the x and y positions of the electrodes selected from the extracellular footprint according to waveform amplitude. 9 overlapping data points in the second quadrant appear to be outliers, but are actually very close in value to the actual angle (all actual angles = $-180°$, all angles predicted by the model = $179.95°$). The geometric mismatch is due to the circular nature of angles. The 26 largest-amplitude electrodes were selected from 144 available electrodes in each extracellular footprint. Their x and y co-ordinates were first normalized by subtracting their respective smallest absolute values. Each set of co-ordinates was labeled with the x and y projections of a unit vector oriented in the direction of the proximal axon (i.e., $cos\,\theta$, and $sin\,\theta$, where θ is the orientation of the proximal axon), and used to independently train two wide neural network regression models. The performance of the models was tested on a held-out data set. The predicted x and y values were used to compute the four quadrant inverse tangent in the closed interval $[-180, 180]$, i.e., the predicted orientation. Actual and predicted orientation in degrees of the proximal axon for a ball-and-stick model is shown along with a null-error diagonal line.

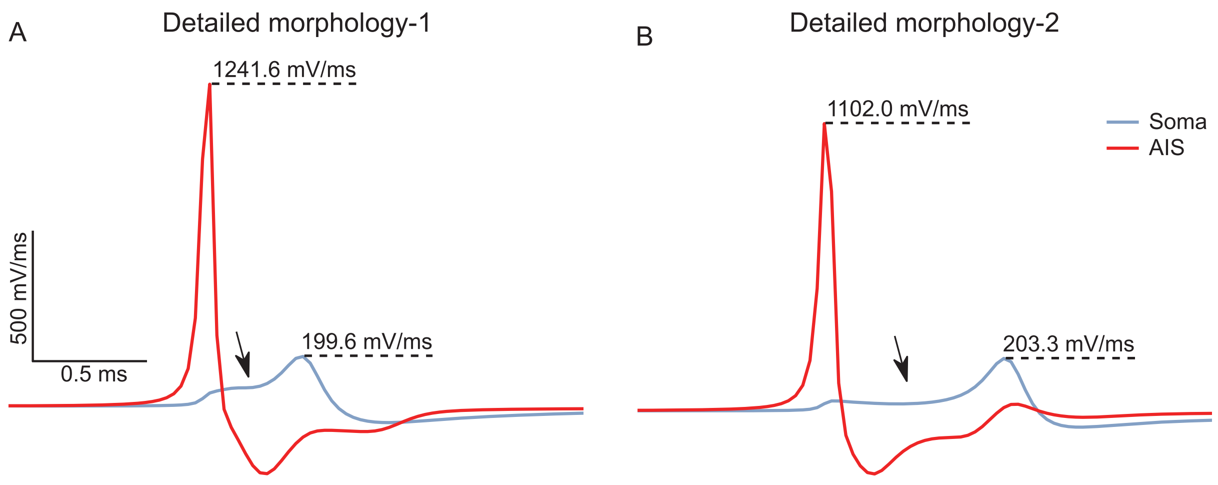

Figure S2. Derivatives of the intracellular voltage traces computed at the soma (blue) and the AIS (red) in the detailed morphology-1 **(A)** and detailed morphology-2 **(B)** models. In both models, the onset of transmembrane currents was earlier at the AIS, where the peak inward current was also significantly larger (A: $1241.6\,\mathrm{mV/ms}$ and $199.6\,\mathrm{mV/ms}$ at the AIS and soma, respectively. B: $1102.0\,\mathrm{mV/ms}$ and $203.3\,\mathrm{mV/ms}$ at the soma and AIS, respectively). In both models, the soma traces show a characteristic "kink" during the depolarization [arrows in **(A)** and **(B)**].

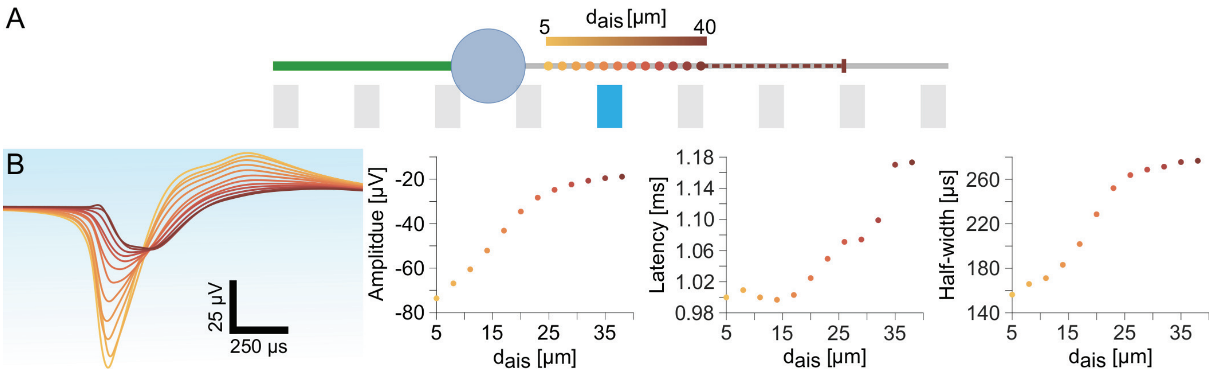

Figure S3. Non-normalized waveform features in the extracellular footprint for the ball-and-stick model as the AIS relocated away from the soma. **(A)** Schematic representation of the ball-and-stick model (green: dendrite, blue: soma, grey: axon) with varying AIS locations (dots along the axon, the colors represent the distance between the proximal AIS and the soma) on the HD-MEA (grey and blue rectangles). The dashed line along the axon represents the extent of the AIS starting at the most distal AIS position. **(B)** The extracellular waveforms at a selected electrode [blue in **(A)**] are shown for various AIS positions in the left-most panel. The color of the traces represent the AIS starting positions indicated as dots along the axon in **(A)**. The amplitude of the extracellular waveforms decreased, as the AIS moved away from the selected electrode. At the same time, the delay (latency to the negative peak) increased, and the spike shapes broadened.

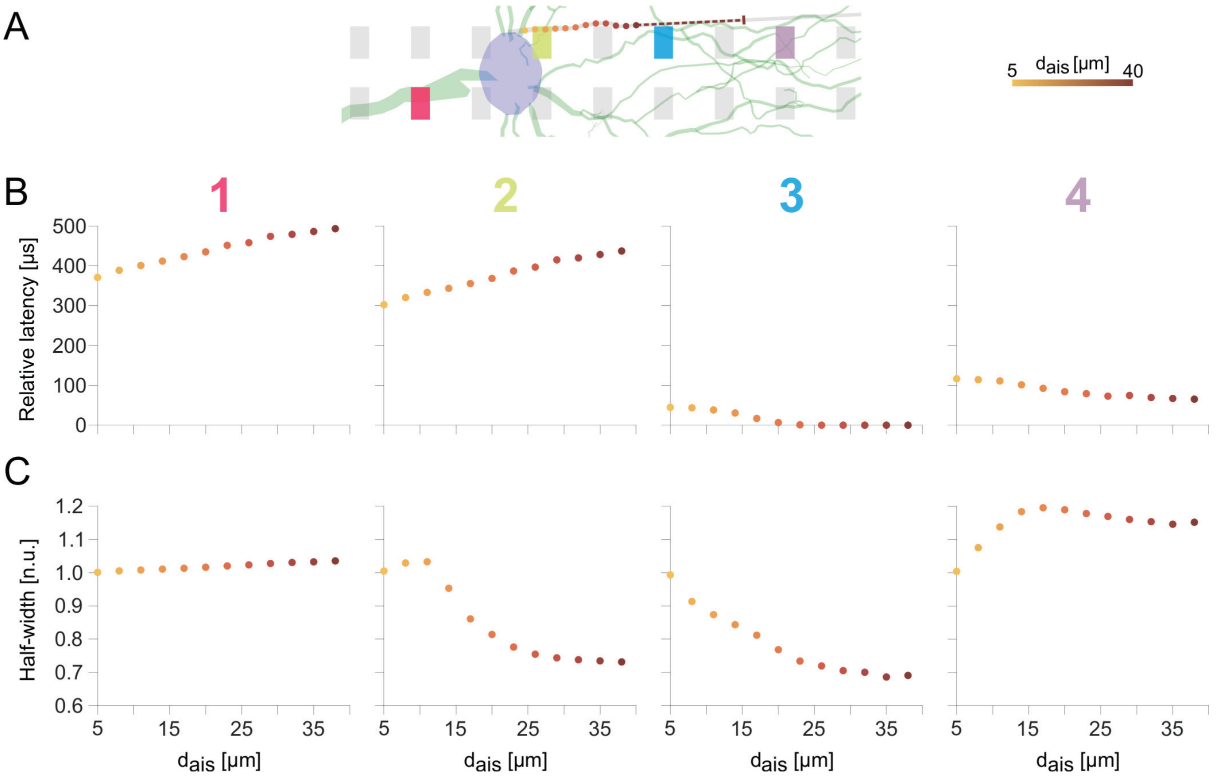

Figure S4. Systematic changes in relative latencies and half-widths were observed in dependence of an AIS relocation in the detailed morphology-1 model. **(A)** Dendrites (green), soma (blue) and axon (gray) are shown as a schematic representation of the modeled cell on the simulated HD-MEA (rectangles). Proximal AIS locations, AIS distances and AIS length were represented as in Figure S3. Features at four selected electrodes (colored) are shown in (B) and (C). Each column in (B) was numbered in the color corresponding to the electrode at which it was computed. **(B)** As the AIS moved away from the soma, the negative peaks, detected at the dendrite (pink, 1) and soma (green, 2), were increasingly delayed. However, at the blue and purple electrodes, towards which the AIS relocated, lower latencies were obtained. All latencies were computed relative to the earliest detected signal peak for each AIS position. **(C)** Distal relocation of the AIS slightly broadened the spike waveform at the dendrite (pink) and distal axon (purple). Close to the soma (green) and in the proximal axon (blue), half-widths generally decreased upon AIS relocation, while a slight increase was observed in the distal axon (purple). Half-widths were normalized by the value obtained at each electrode for the initial AIS position.

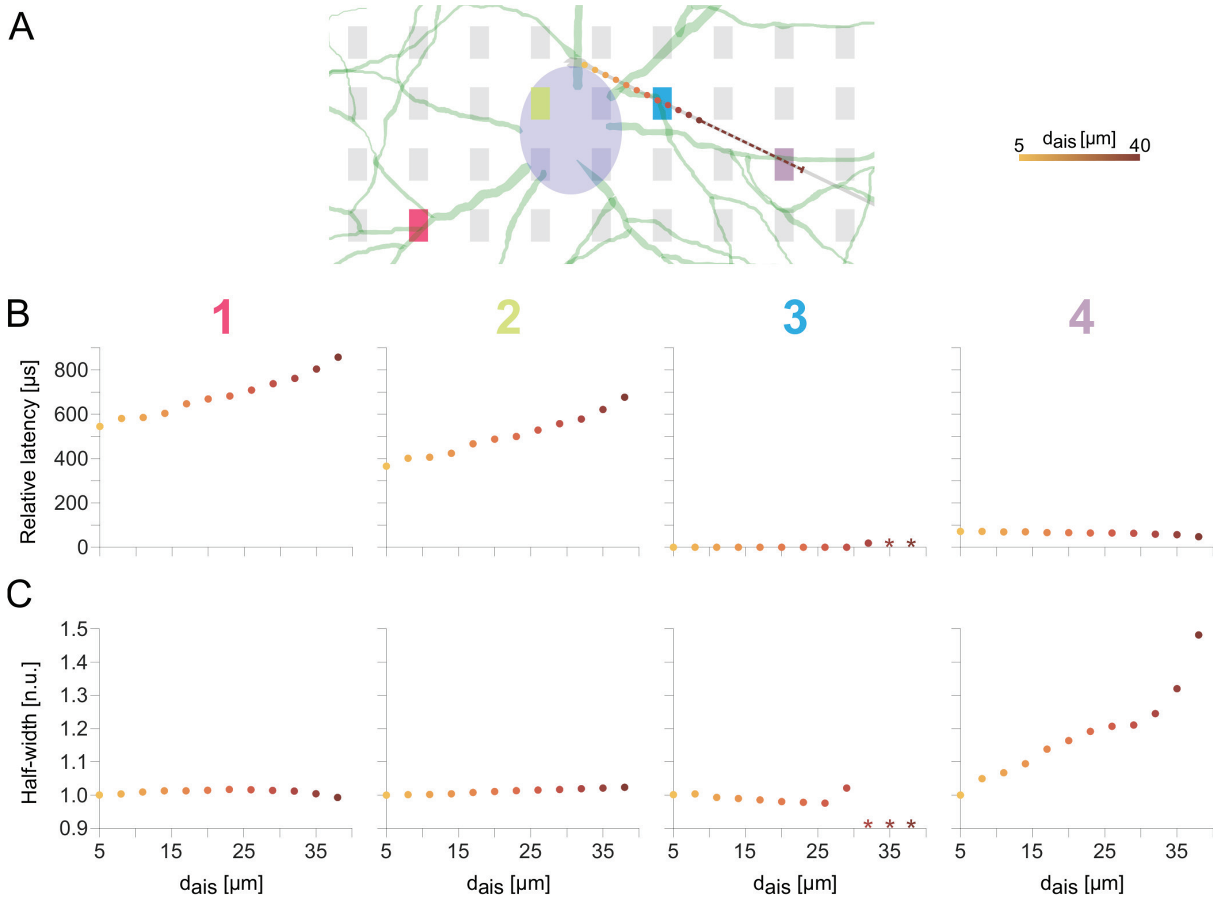

Figure S5. Changes in spike waveform features in dependence of AIS relocation in the detailed morphology-2 model. **(A)** Schematic representation of the detailed morphology-2 model on a simulated HD-MEA. Proximal AIS locations, AIS distances and AIS length was represented as in Figure S3. Features at four selected electrodes (colored) are shown in columns of (B) and (C). **(B)** As in Figure S4, delayed peaks were observed at the electrodes in close proximity to the dendrite (pink) and the soma (green), as the AIS moved away from those. The spike recorded at the blue electrode was for almost all AIS positions the earliest (zero relative latency). All latencies were computed relative to the earliest detected signal peak for each AIS position. **(C)** A relative broadening of spike waveforms was observed only at the purple electrode. Note: Feature values could not be reliably computed for a couple of AIS positions at the blue electrode due to the complexity of the corresponding spike shapes (see waveforms in Figure 4B). They were set to zero and marked with stars in the feature plots.

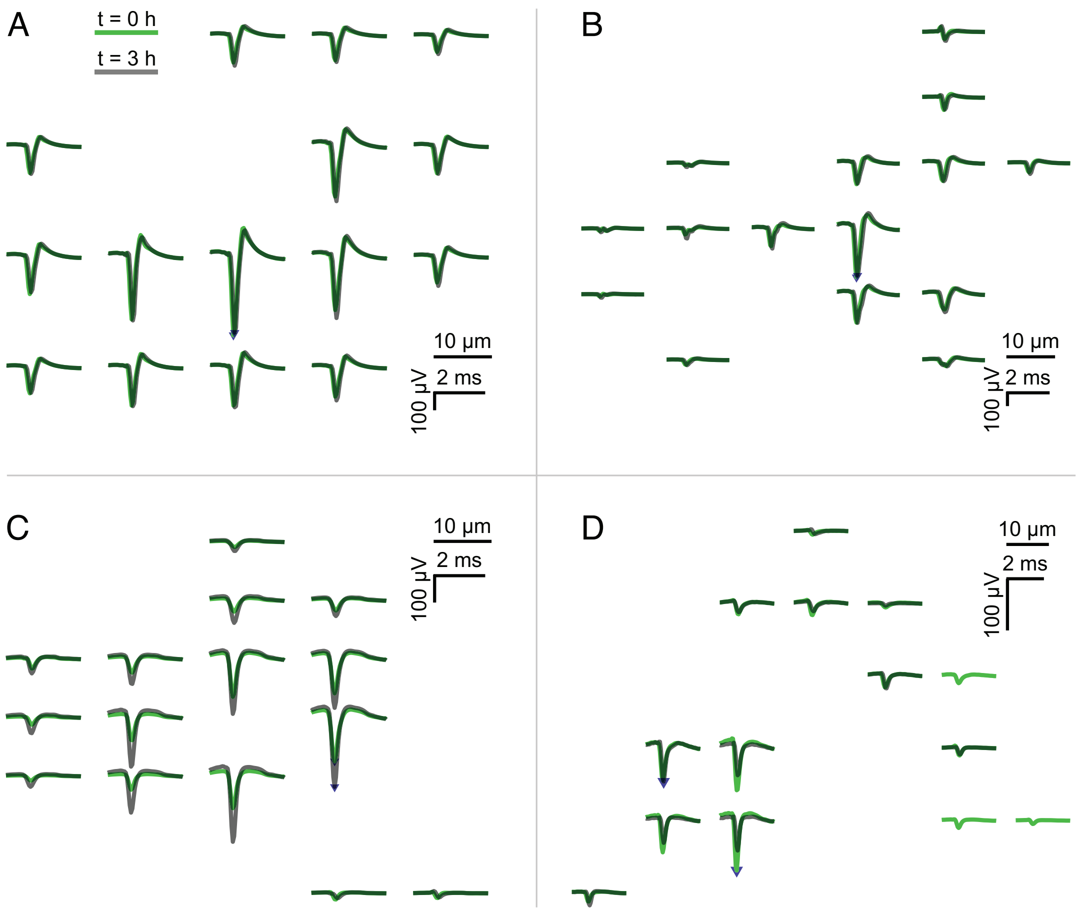

Figure S6. **(A-D)** The extracellular electrical footprints (templates) of four putative neurons (units) from primary dissociated rat cortex, cultured on HD-MEAs and recorded 3 h apart at 14 days in vitro. Spike sorting was performed on each $15\,\mathrm{min}$-long recording acquired from 817 electrodes. Unit templates recovered from the first recording (t=$0\,\mathrm{h}$, green) were spatiotemporally correlated with those from the second (t=$3\,\mathrm{h}$, gray) recording. Template pairs with the highest correlation scores were matched, and their footprints were overlaid. Overall, we were able to recover 230 well matched units that were stable in terms of waveform features. In some units, noticeable changes in waveform amplitudes were observed (like in C-D) and could indicate slight shifts in the neuronal position relative to the electrodes.
